# Recent Progress in Single‐Crystalline Perovskite Research Including Crystal Preparation, Property Evaluation, and Applications

**DOI:** 10.1002/advs.201700471

**Published:** 2017-11-10

**Authors:** Yucheng Liu, Zhou Yang, Shengzhong (Frank) Liu

**Affiliations:** ^1^ Key Laboratory of Applied Surface and Colloid Chemistry National Ministry of Education School of Materials Science and Engineering Shaanxi Normal University Xi'an 710119 China; ^2^ Shaanxi Key Laboratory for Advanced Energy Devices School of Materials Science and Engineering Shaanxi Normal University Xi'an 710119 China; ^3^ Shaanxi Engineering Lab for Advanced Energy Technology School of Materials Science and Engineering Shaanxi Normal University Xi'an 710119 China; ^4^ Institute for Advanced Energy Materials School of Materials Science and Engineering Shaanxi Normal University Xi'an 710119 China; ^5^ iChEM Dalian National Laboratory for Clean Energy Chinese Academy of Sciences Dalian 116023 China; ^6^ Dalian Institute of Chemical Physics Chinese Academy of Sciences Dalian 116023 China

**Keywords:** crystal growth, optoelectronic applications, perovskite, single crystal

## Abstract

Organic–inorganic lead halide perovskites are promising optoelectronic materials resulting from their significant light absorption properties and unique long carrier dynamics, such as a long carrier lifetime, carrier diffusion length, and high carrier mobility. These advantageous properties have allowed for the utilization of lead halide perovskite materials in solar cells, LEDs, photodetectors, lasers, etc. To further explore their potential, intrinsic properties should be thoroughly investigated. Single crystals with few defects are the best candidates to disclose a variety of interesting and important properties of these materials, ultimately, showing the increased importance of single‐crystalline perovskite research. In this review, recent progress on the crystallization, investigation, and primary device applications of single‐crystalline perovskites are summarized and analyzed. Further improvements in device design and preparation are also discussed.

## 1 Perovskite Materials

**Figure 1:**
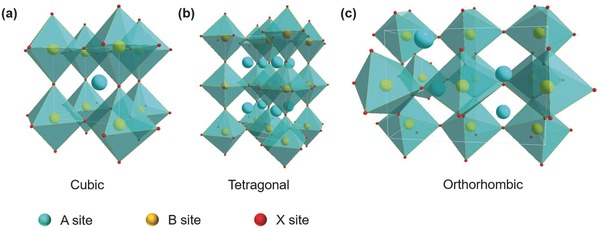
The crystal structures of perovskite materials with different symmetries: a) cubic phase, b) tetragonal phase, and c) orthorhombic phase.

The mineral CaTiO_3_ was first discovered by Russian scientist Gustav Rose in 1839 and named perovskite after the Russian mineralogist, Count Lev Aleksvich Von Perovski. Since then, materials with the formula ABX_3_ and similar crystal structures as CaTiO_3_ are defined as perovskites. Depending on the ionic or elemental radii of the A, B, and X elements, the crystal structure could change from a high‐symmetry cubic phase to a tetragonal phase or to a low‐symmetry orthorhombic phase (**Figure** **1**).^1^, ^2^, ^3^ The variable chemical composition and tunable crystal structure endow perovskites with versatile properties, such as magnetism,^4^, ^5^ ferroelectricity,^6^, ^7^ high ion conductivity,^8^, ^9^ and various applications in magnets, memories, solid oxide fuel cells, etc.

Organic groups, such as CH_3_NH_3_^+^ (MA) and CH_7_N_2_^+^ (FA), having similar sized inorganic ions, can also form perovskite materials, called organic–inorganic hybrid perovskites. The organic group size will determine whether the crystal structure is 3D or 2D. In this review, unless otherwise specified, perovskite represents an inorganic–organic hybrid perovskite. These materials have been investigated since the 1970s,^10^, ^11^ and perovskite thin films with outstanding optoelectronic performances were first fabricated by Mitzi et al. using a one‐step method.^12^, ^13^, ^14^, ^15^, ^16^, ^17^ However, in 2009, pioneering work utilizing CH_3_NH_3_PbI_3_ and CH_3_NH_3_PbBr_3_ as absorbers in sensitized solar cells brought them into the scientific spotlight.^18^

Furthermore, additional optoelectronic properties have been discovered, including long balanced hole–electron diffusion length,^19^, ^20^, ^21^, ^22^, ^23^ high carrier mobility,^21^, ^24^ high absorption coefficient in visible region,^25^, ^26^ and long carrier lifetime.^21^, ^22^, ^25^, ^27^, ^28^, ^29^ These optoelectronic properties make hybrid perovskites ideal candidates for solar cell applications,^22^, ^25^, ^30^, ^31^, ^32^, ^33^, ^34^, ^35^, ^36^, ^37^, ^38^ LEDs,^39^, ^40^, ^41^ lasers,^42^, ^43^ etc. A certificated power conversion efficiency (PCE) as high as 22.1% has been achieved, which approaches the highest PCE for Si solar cells.^44^ Although many efforts have been made to disclose the amazing optoelectronic properties, there is still some debate on the origin of the current density–voltage (*J*–*V*) curve hysteresis,^45^, ^46^, ^47^ defect‐induced ion migration,^48^, ^49^, ^50^, ^51^ grain boundaries,^46^, ^52^ unbalanced electron–hole extraction,^37^, ^53^ etc. To fully obtain and understand the intrinsic characteristics of perovskites, high‐quality single‐crystalline samples are needed.

Over the last 2 years, an increasing number of researchers have begun studying single‐crystalline perovskites, including crystal preparations,^21^, ^27^, ^28^, ^43^, ^54^, ^55^, ^56^, ^57^, ^58^, ^59^, ^60^, ^61^, ^62^, ^63^, ^64^, ^65^ property studies,^21^, ^27^, ^66^, ^67^, ^68^, ^69^, ^70^, ^71^, ^72^, ^73^, ^74^, ^75^, ^76^, ^77^, ^78^, ^79^, ^80^ and applications.^56^, ^81^, ^82^, ^83^, ^84^, ^85^, ^86^, ^87^, ^88^, ^89^, ^90^, ^91^ For example, an extremely long carrier lifetime (up to 20 µs) and carrier diffusion length (175 µm) have been found,^21^ which has received a great deal of attention. In this review, we will briefly summarize single‐crystalline perovskites, including crystal growth, basic properties, and applications, followed by a discussion of their challenges and opportunities.

## 2 Crystal Growth, Optoelectronic Properties, and Applications

In this section, the crystal growth strategies and basic principles for single‐crystalline perovskites and single‐crystalline thin films are summarized. Furthermore, the optoelectronic properties and corresponding applications of perovskite crystals are included and discussed, which includes properties in optics, carrier dynamics and applications in solar cells, photodetectors, and lasers.

### 2.1 Crystal Growth

Methods used to grow single crystals are classified by growth environment and include solid growth, liquid growth, and vapor growth. To date, all reported perovskite crystals have been prepared with the liquid growth method.^21^, ^27^, ^28^, ^55^, ^61^ Generally, crystals will form by slowly reducing the solubility of the target samples in a precursor solution. To produce perovskite single crystals, three strategies have been used to modulate solubility: cooling HX‐based solutions (use of HCl, HBr, and HI as the solvent), heating *N*,*N*‐dimethylformamide (DMF)/γ‐butyrolactone (GBL)/dimethyl sulfoxide (DMSO)‐based solutions, and an antisolvent method.

#### 2.1.1 Cooling HX‐Based Precursor Solutions

**Figure 2:**
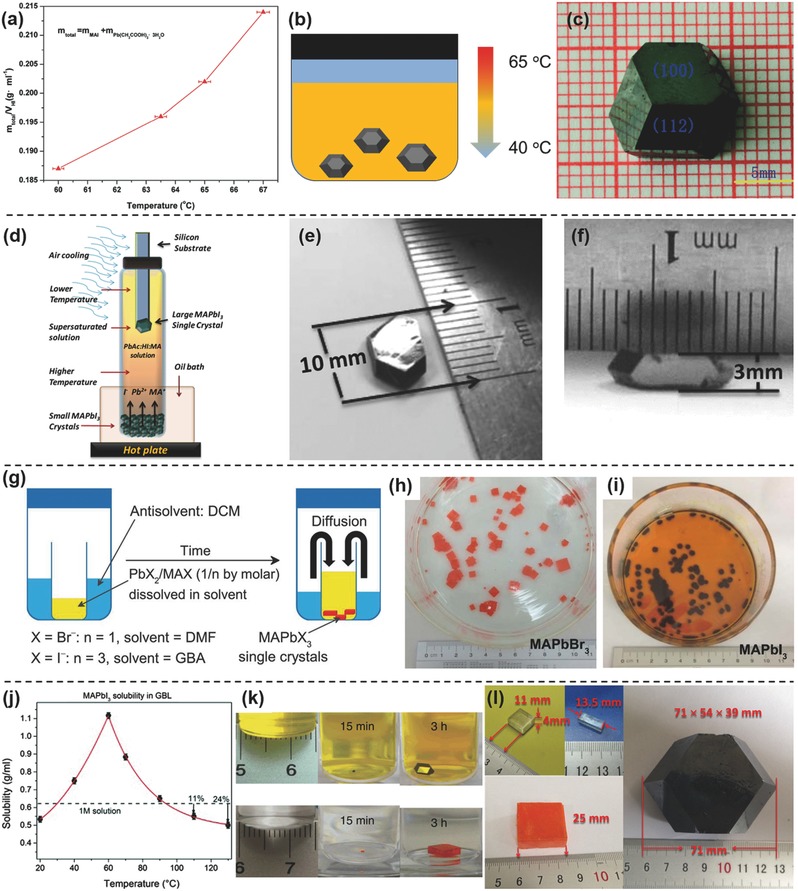
Methods used for single‐crystal preparation and the basic theories behind these methods: a–c) low temperature‐induced single‐crystal growth during which the solubility of the perovskite will decrease with a lower solution temperature; d–f) schematic illustration of TSSG method and the single crystal obtained by this method; g–i) AVC method to prepare single‐crystalline perovskites and their shape and size recorded by a camera; j) the solubility curve of CH_3_NH_3_PbI_3_ in GBL, which decreased with temperature at temperatures over 60 °C; k) the single‐crystalline perovskites obtained quickly with an ITC) method, which is based on the solubility curve in (j); l) a large single‐crystalline perovskite was prepared by repeating the ITC process several times. a–c) Reproduced with permission.^61^ Copyright 2015, RSC. d–f) Reproduced with permission.^11^ Copyright 2015, AAAS. g–i) Reproduced with permission.^27^ Copyright 2015, AAAS. j) Reproduced with permission.^57^ Copyright 2015, RSC. k) Reproduced with permission.^28^ Copyright 2015, published under the terms of CC‐BY 4.0 license. l) Reproduced with permission.^55^

The perovskite solubility in a HX‐based solvent will decrease with a lower solution temperature (**Figure** **2**a).^61^ Following this basic principle, cooling the precursor can produce single‐crystalline perovskites, which was first reported by Poglitsch and Weber more than 20 years ago.^3^ Briefly, cooling a concentrated aqueous solution of HX acid, Pb^2+^ and CH_3_NH_3_^+^ from ≈100 °C to room temperature, allows for MAPbX_3_ (X = Cl, Br, I) crystals to be obtained. Except for MAPbI_3_, the temperature should be controlled above a temperature of 40 °C,^92^ otherwise, a colorless MA_4_PbI_6_·2H_2_O will be formed. Without careful control of the crystal growth process, it is hard to obtain large and regular‐shaped crystals. Recently, this method has been modified and used to grow larger crystals with a regular shape.^21^, ^61^

The Huang group applied a top‐seeded solution growth (TSSG) to prepare large single crystals (Figure 2d–f).^21^ First, many small crystals were collected by lowering the saturated perovskite solution and placing it at the bottom of the growth solution, which was kept at 75 °C. A Si wafer was then immersed into the precursor solution. Due to the lower temperature at the top of the vessel (shown in Figure 2d), small crystals will form on the Si wafer. With the concentration higher at the bottom, there will be convection between the bottom and top of the solution, which will provide enough material for single‐crystal growth. For large crystal growth, only one seed crystal was left, which will continue to increase in size by extending the growth process. Finally, a crystal larger than 10 mm was obtained. By alternating the CH_3_NH_2_ and HI blending solution with MAI, the Tao group also produced large crystals with sizes exceeding 10 mm (Figure 2a–c).^61^ However, the growth speed is very slow using this method, with taking months to obtain 10 mm crystals. Recently, the Yan group found that CH_3_NH_3_Cl could reduce the solubility of MAPbI_3_ in an HI‐based solution and accelerate the crystal growth speed.^93^ In 5 d, a 20 mm × 18 mm × 6 mm crystal was produced without sacrificing crystallinity, carrier mobility and carrier lifetime. Other kinds of perovskites, such as MAPbBr_3_, MAPbCl_3_, MAPb(Cl*_x_*Br_1−_ *_x_*)_3_, MASnI_3_, and FAPbI_3_, have also been successfully prepared by this method.^94^, ^95^, ^96^, ^97^, ^98^

#### 2.1.2 Antisolvent Diffusion

In 2014, the Cheng and Seok groups found antisolvent‐assisted crystalline methods, which are called the fast crystallization deposition^99^ and solvent engineering^100^ approaches to produce dense and high‐quality perovskite films, respectively. Both of these methods are based on a similar principle‐different solubility of MAPbX_3_ (X = Cl, Br, I) in different solvents.^99^, ^100^, ^101^, ^102^, ^103^, ^104^, ^105^ For example, hybrid halide perovskites show good solubility in DMSO, DMF, and GBL, while they show non or low solubility in chlorobenzene, benzene, diethyl ether, etc. The antisolvent could accelerate the crystallization of perovskite during spin coating and facilitate uniform film formation. Using this same strategy, the Bakr group developed a new method to grow large MAPbBr_3_ and MAPbI_3_ crystals called the antisolvent vapor‐assistant crystallization (AVC) method (Figure 2g).^27^ Taking MAPbBr_3_ as an example, PbBr_2_ and MABr powders (molar ratios of 1:1) were dissolved in DMF to form the precursor solution. The solution and dichloromethane (DCM) were then sealed in a closed vessel and stored. The DCM will slowly evaporate, and its vapor will diffuse into the precursor solution because DCM mixes well with DMF. The diffusion of DCM in DMF will consume some DMF solvent. Since PbBr_2_ and MABr cannot dissolve in DCM, the consumption of DMF by DCM will increase the concentration of MABr and PbBr_2_ in the solution. This will result in the formation of MAPbBr_3_ crystals. Recently, the Huang group found that it was difficult to get high‐quality MAPbBr_3_ with a 1:1 mole ratio of MABr and PbBr_2_ because of their different solubility's in DMF. The PbBr_2_ and MABr molar ratio of 0.8 can produce high‐quality crystals with higher carrier mobility and lifetime.^106^

#### 2.1.3 Inverse Temperature Crystallization (ITC)

Large perovskite crystals can be quickly produced by increasing the temperature of the DMF/DMSO/GBL precursor solution, which results from the abnormal solubility of the perovskite in these solvents. Figure 2i shows that the solubility of MAPbI_3_ in GBL drops almost twofold as the temperature of the solution increased from 60 to 100 °C.^57^ Following the solubility curve, the highest concentration of MAPbI_3_ in the solution could be controlled by adjusting the temperature. During the heating of the saturated precursor solution, perovskite crystals will precipitate at the bottom of the solution since the precursor concentration is larger than what the solubility allows. This method quickly produced high‐quality perovskite crystals, with a 5 mm‐sized crystal obtained in 3 h.^28^ Recently, the role of surface tension in the rapid synthesis of metal halide perovskites by ITC has been uncovered by Bakr et al.^107^ After a dedicated investigation, our group further found that a specific solvent works best for a particular halide perovskite. For example, DMSO works best for MAPbCl_3_, DMF works best for MAPbBr_3_ and GBL works best for MAPbI_3_.^55^ If DMSO was used for MAPbI_3_, it was difficult to obtain MAPbI_3_ crystals. Furthermore, by repeating and carefully controlling the ITC process for several times, 70 mm‐sized perovskite single crystals could be obtained (as seen in Figure 2l). More recently, our group has reported that the crystal size further exceeds 120 mm.^108^

#### 2.1.4 Geometry‐Controllable Perovskite Crystals

**Figure 3:**
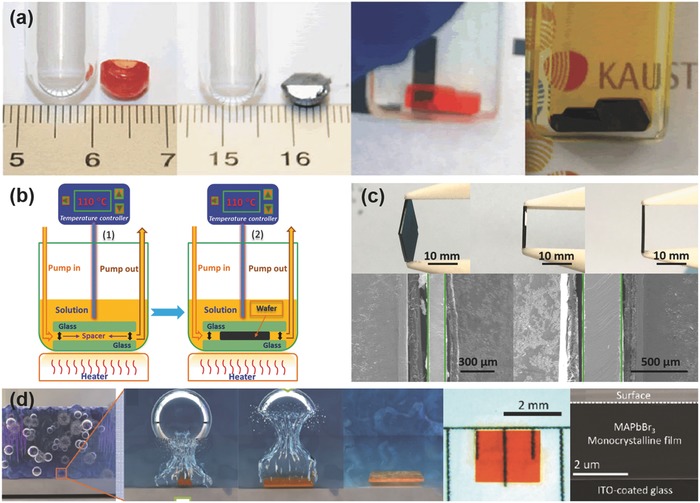
The strategies used to modulate the shape and thickness of single‐crystalline perovskites and the corresponding crystals obtained by these methods: a) sphere‐shaped and stair‐shaped perovskite prepared using a round‐bottom test tube and a 2 mm curette; b) schematic illustration of the method to control and adjust the thickness of the perovskite wafers; c) pictures of the perovskite wafers with thicknesses from 150 µm to ≈mm;^109^ and d) a CTAC strategy to prepare a single‐crystalline film at the µm scale. a) Reproduced with permission.^28^ Copyright 2015, published under the terms of CC‐BY 4.0 license. b,c) Reproduced with permission.^109^ d) Reproduced with permission.^56^

Beyond regular‐shaped perovskite crystals, thin‐plate, column‐shaped, and pillar arrays have been made. These are more important for device applications than bulk crystals and have been produced using templates by confining the crystal growth along specific directions (**Figure** **3**a,b). The Bakr group reported a round shape and stair‐shaped perovskite crystals via a round‐bottom test tube and a 2 mm curette as templates.^28^ Our group successfully made thickness‐tunable perovskite wafers by confining the crystal growth along the *z* direction (Figure 3b). The thickness of single‐crystalline wafers could be adjusted from ≈mm to 150 µm.^109^ Using the same strategy, the Wan and Kuang groups obtained extremely thin perovskite wafers approximately 10 µm.^62^, ^86^, ^110^ Alternatively, to a rigid template, a soft template has also been used to modulate the crystal growth.^111^ Tuning the wettability of the precursor, a liquid knife method has been proposed to prepare high‐quality perovskite crystal arrays on the substrate.^43^ Amazingly, thin perovskite crystals with micrometer thicknesses were successfully produced by a cavitation‐triggered asymmetrical crystallization (CTAC) strategy, generated by a high powered ultrasonic machine.^56^

Except organic–inorganic lead halide perovskites, inorganic lead halide perovskites (CsPbBr_3_,^112^, ^113^, ^114^, ^115^ Cs_2_Pb_1−_ *_x_*Bi*_x_*Br_6_),^116^ lead free perovskites (CH_3_NH_3_CaI_3_,^117^ CH_3_NH_3_SnI_3_,^96^ NH(CH_3_)_3_SnBr_3_,^118^ NH(CH_3_)_3_SnCl_3_,^118^ Cs_2_AgBiBr_6_,^119^ Cs_2_PdBr_6_)^120^ have also been prepared using a similar method as discussed above. The inorganic lead halide perovskite CsPbBr_3_, which possesses higher thermal stability, could also been fabricated using the Bridgman method, with the highest furnace temperature approximately 1470 °C. The obtained CsPbBr_3_ shows unprecedent carrier mobility as high as 2290 cm^2^ V^−1^ s and a trap density as low as 1.9 × 10^9^ cm^−3^, demonstrating its high electronic quality and crystal quality.

### 2.2 Optoelectronic Properties

Knowing the basic properties of a semiconductor material, such as carrier concentration, trap position and density, carrier mobility, carrier lifetime, diffusion length, is a primary step before designing a suitable application for it. This also holds true for perovskite crystals. All optoelectronic properties indicate that a single‐crystalline perovskite is much better than its thin‐film counterpart because there are many grain boundaries and defects in the polycrystalline film. Details on the basic optoelectronic properties could be found in the following three sections and **Table** **1**.

**Table 1:** The critical optoelectronic parameters of single‐crystalline perovskite reported in the literature

Materials	Band gap or absorption onset	Mobility [cm^2^ V^−1^ s^−1^]	Conductance [Ω^−1^ cm^−1^]	Carrier lifetime and method	Carrier concentration and type [cm^−3^] (n or p)	Diffusion length	Trap density [cm^−3^]
MAPbBr_3_ 27	2.21 eV 570 nm	115 (TOF) 20–60 (Hall) 38 (SCLC)		τ_s_ = 41 ns τ_b_ = 457 ns (PL)	5 × 10^9^ to 5 × 10^10^ p	3–17 µm	5.8 × 10^9^
MAPbI_3_ 27	1.51 eV 821 nm	2.5 (SCLC)	10^−8^	τ_s_ = 22 ns τ_b_ = 1032 ns PL	2 × 10^10^	2–8 µm	3.3 × 10^10^
MAPbBr_3_ 28	2.18 eV 574 nm	24 (SCLC)		τ_s_ = 28 ns τ_b_ = 300 ns PL		1.3–4.3 µm	3 × 10^10^
MAPbI_3_ 28	1.51 eV 820 nm	67.2 (SCLC)		τ_s_ = 18 ns τ_b_ = 570 ns PL		1.8–10.0 µm	1.4 × 10^10^
MAPbI_3_ 21	850 nm	164 ± 25 Hole mobility (SCLC) 105 Hole mobility (Hall) 24 ± 6.8 electron SCLC		82 ± 5 µs TPV 95 ± 8 µs impedance spectroscopy (IS)	9 × 10^9^ p	175 ± 25 µm	3.6 × 10^10^ for hole 34.5 × 10^10^ for electron
MAPbI_3_ 55	1.53 eV 784 nm	34 Hall			8.8 × 10^11^ p		1.8 × 10^9^ for hole 4.8 × 10^10^ for electron
MAPbBr_3_ 55	2.24 eV 537 nm	4.36 Hall			3.87 × 10^12^ p		2.6 × 10^10^ for hole 1.1 × 10^11^ for electron
MAPbCl_3_ 55	2.97 eV 402 nm	179 Hall			5.1 × 10^9^ n		
MAPbCl_3_ 68	2.88 eV 440 nm	42 ± 9 (SCLC)	2.7 × 10^−8^	τ_s_ = 83 ns τ_b_ = 662 ns PL	4.0 × 10^9^ p	3.0–8.5 µm	3.1 × 10^10^
FAPbI_3_ 122	1.49 eV 870 nm	40 ± 5 Hole mobility SCLC	1.8 × 10^−8^		2.8 × 10^9^		1.34 × 10^10^

#### 2.2.1 Electronic Properties

**Figure 4:**
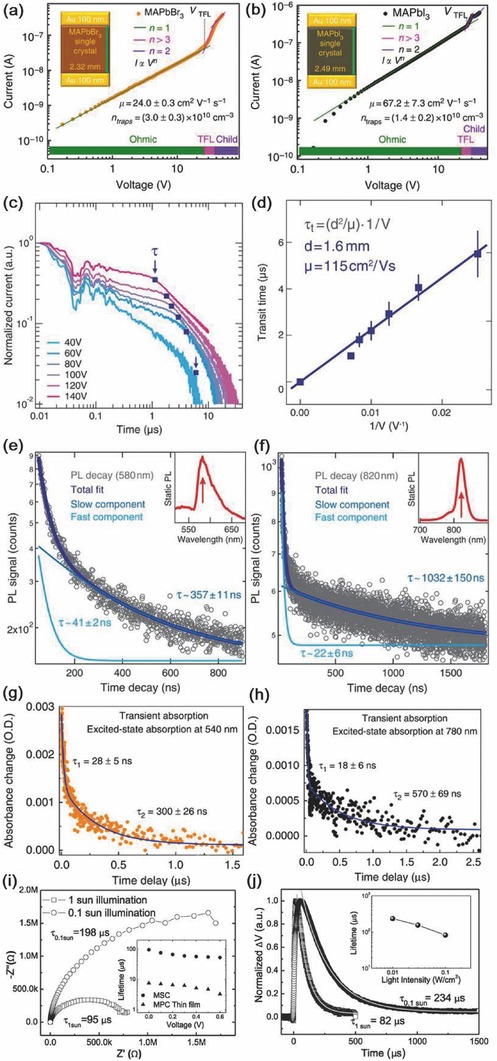
Methods used to investigate the carrier transport and dynamic parameters and corresponding results: a,b) trap density, carrier mobility, and carrier concentration of the crystal disclosed using the SCLC method; c,d) TOF method and corresponding results revealing the carrier mobility of a single‐crystalline perovskite; carrier lifetime of perovskites disclosed by different ways: e,f) photoluminescence decay; g,h) transient absorption; and i,j) impedance and transient photovoltage decay methods. a,b,g,h) Reproduced with permission.^28^ Copyright 2015, Nature Publishing Group. c–f) Reproduced with permission.^27^ Copyright 2015, AAAS. i,j) Reproduced with permission.^21^ Copyright 2015, AAAS.

The carrier mobility reflects the factors that hinder carrier transportation, which is governed by the mean free time during carrier movement and effective mass of the carrier as expressed in Equation (1),

(1) $$\mu \;\;=\;\;\frac{q\tau }{m*}$$

where τ is the mean free time and m* is the effective mass. Various methods have been utilized to investigate carrier mobility of perovskite single crystals. Space charge limited current (SCLC),^21^, ^27^ time of flight (TOF),^21^ and Hall effect^21^, ^55^ methods give different values, which are summarized in Table 1. The difference may be the result of different transport processes measured by these methods.^121^ The trap density of a single crystal is ≈10^11^ to ≈10^9^ cm^−3^ (Table 1 and **Figure** **4**a–d), approximately 4–5 orders of magnitude lower than that of a polycrystalline perovskite film.^21^, ^27^, ^28^, ^93^ The mean free time τ of the carriers in a single crystal will be longer because it possesses high crystalline quality with fewer defect and traps. The carrier mobility of a single crystal should be larger due to less scattering during charge transportation. As shown in Table 1, the highest carrier mobility of MAPbBr_3_ and MAPbI_3_ can reach 115 and 164 cm^2^ V^−1^ s^−1^, respectively.

#### 2.2.2 Carrier Dynamics

Carrier lifetime is an important factor that should be considered when designing a device, especially a solar cell. After being excited by photons, a material will be in an excited state. The excess free holes and electrons will recombine back to the ground state. Generally, the recombination process could be classified as radiative and nonradiative. During radiative recombination, the hole and the electron will recombine with each other and emit a photon. While in a nonradiative recombination process, the trap will induce the carrier recombination without photon emission. The carrier lifetime determined by these two processes is expressed as^70^

(2) $$\frac{1}{\tau }\;\;=\;\;\frac{1}{\tau_{r}}\;\;+\;\;\frac{1}{t_{\mathrm{nr}}}$$

where τ, τ_r_, and τ_nr_ are the carrier lifetime, radiative lifetime, and nonradiative lifetime, respectively. Here, τ_r_ is an intrinsic parameter, which will not be affected. However, τ_nr_ is highly related to the trap density and could be tuned by quality control. As the trap gets smaller, τ becomes closer to τ_r_. As shown in Figure 4e–j, photoluminescence decay,^27^ transient absorption,^28^ transient photovoltage decay,^27^, ^85^ and impedance^27^ methods have been used to examine carrier lifetime in single‐crystalline perovskite. The photoluminescence (PL) decay and transient absorption gives a lifetime of nearly 1000 ns.^27^ However, the transient photovoltaic (TPV) and impedance tests, based on the device structured sample (Au/perovskite/Ga), delivered extremely long carrier lifetimes exceeding 80 µs, which is almost 100 times larger than the PL lifetime.^21^, ^93^ The long carrier lifetime indicates a small amount of trap state existing in single‐crystalline perovskite.

The carrier diffusion length is determined by the carrier mobility and carrier lifetime, as shown in Equation (3), 21, 23, 27

(3) $$L_{D}\;\;=\;\;\sqrt{kT\frac{\mu \tau }{q}}$$

where *k* is the Boltzmann constant, *T* is the absolute temperature, and *q* is the element charge. As predicted by this equation, higher mobility and longer carrier lifetimes will yield a longer carrier diffusion length, which is important for solar cell applications. As summarized in Table 1, when the photoluminescence lifetime was used to calculate the diffusion length, the longest diffusion length for MAPbBr_3_ and MAPbI_3_ could reach 17 and 10 µm, respectively,^27^, ^28^ which are much longer than those of their thin‐film counterparts. The Huang group also tested the carrier diffusion length in a solar cell device. By using TPV and impedance methods to investigate the carrier lifetime under 1 sun illumination, a 175 µm diffusion length was obtained for single‐crystalline MAPbI_3_.^21^

#### 2.2.3 Optical Properties

**Figure 5:**
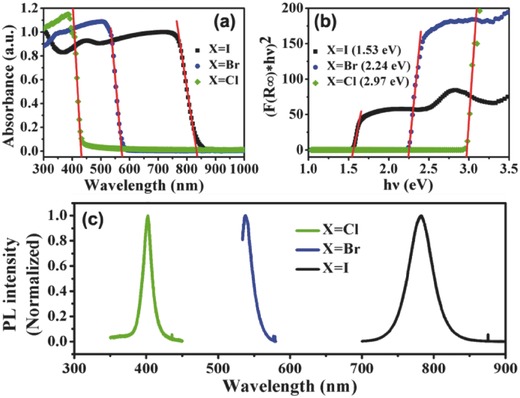
Light absorption and photoemission properties of single‐crystalline perovskites. Reproduced with permission.^55^

Compared to thin films, single crystals show extended light absorption (redshift).^21^, ^27^, ^55^, ^108^ **Figure** **5** shows absorption onsets of MAPbCl_3_, MAPbBr_3_, and MAPbI_3_ shift to 440 nm, 570 nm, and 850 nm, respectively‐approximately 40 nm, 20 nm, and 50 nm shifts relative to their thin‐film counterparts, respectively.^56^ The optical band gaps of MAPbCl_3_, MAPbBr_3_, MAPbI_3_ have also been estimated to be 2.97, 2.24, and 1.53 eV, using Tauc plots (Figure 5b), in which *hν* stands for the photon energy and (*F*(*R*(∞))*hν)^2^ represents the product of the absorption coefficient and photon energy. Much longer light absorption regions could absorb a larger number of photons and may produce higher photocurrents using the same material. Except for the extended absorption region, mix‐halide perovskites have also been successfully prepared, which show tunable light absorption and photoemission across the entire UV–vis–NIR light region.^42^, ^108^, ^123^, ^124^, ^125^ This property is very advantageous to the design of a perovskite‐based display unit, as well as colorful solar panels. The photoluminescence peaks of MAPbCl_3_, MAPbBr_3_, MAPbI_3_ are located at 402, 537, and 784 nm, respectively, which are smaller than the absorption onsets positions. The difference, which may have arose from the direct and indirect band gap profile of perovskite,^126^, ^127^ is stilled under investigation.

### 2.3 Applications

Semiconductor materials have very special optical and electronic properties. They are the base of sensors, detectors, CPUs, etc. Hybrid perovskite possess great optoelectronic properties, as summarized in last section, and shows great potential in solar cells, lasers, photodetectors, etc. The typically applications of these materials are summarized in the following sections.

#### 2.3.1 Solar Cells

**Figure 6:**
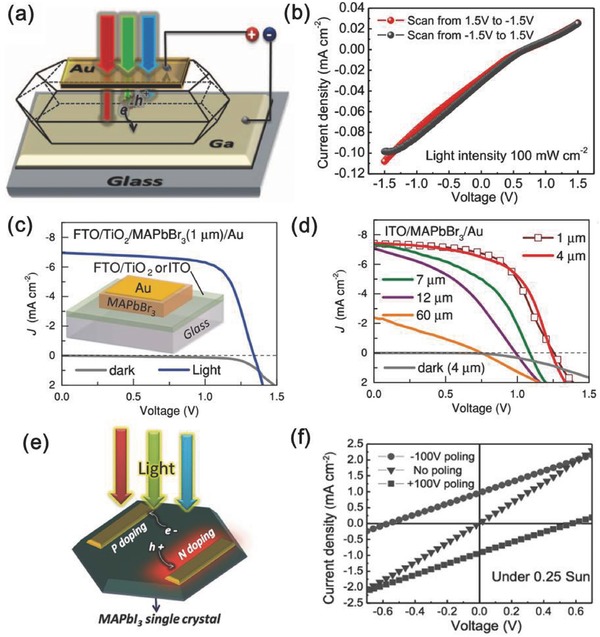
Single‐crystalline perovskite‐based solar cell devices with different configurations: a) thick single‐crystalline perovskite solar cell with a semitransparent Au electrode as the HTL and Ga electrode as the electron transport layer. Reproduced with permission.^21^ Copyright 2015, AAAS. b) *J*–*V* curve of the solar cell shown in figure (a); c) the device structure of a single‐crystalline MAPbBr_3_ film‐based solar cell and its photovoltaic performance. Reproduced with permission.^56^ d) Effect of the single‐crystalline MAPbBr_3_ thickness on the photovoltaic performance of single‐crystalline solar cells; e) schematic illustration of a lateral structured single‐crystalline perovskite solar cell and its photovoltaic performance f). Reproduced with permission.^128^

As a semiconductor material with a large absorption coefficient and long carrier diffusion length, solar cells should be the best model device to show their advantages, which are similar to a perovskite single crystal. However, there are obstacles in direct usage of a large single crystal. The Huang group built a solar cell device, including a semitransparent Au/perovskite/Ga device (**Figure** **6**a,b),^21^ but this delivered minimal photovoltaic response. The main reasons for the low response are that photon‐generated carriers could not be fully collected in a thick device, as well as the high intrinsic resistance of perovskite materials will bring ohmic loss via a thick absorber layer. Thus, the thin single‐crystalline films, in which photon‐generated carriers will transport a short length, are urgently needed to build a single‐crystalline perovskite solar cell, which shows the potential use of single‐crystalline materials.

The Bakr group developed a CTAC strategy (Figure 3d) to prepare thin single‐crystalline perovskite films at the µm scale.^56^ The solar cell with 1 µm‐thick single‐crystalline films showed the best photovoltaic performance (Figure 6c,d). Increasing the absorber thickness from 4 to 10 µm will increase the serial resistance of the device and reduce the FF. When a 60 µm‐thick single‐crystalline film was used, the *J*_sc_, *V*_oc_, and FF decreased dramatically because the charge carrier cannot be collected efficiently. Recently, using space confine strategy, the Kuang group successfully obtained a microscale MAPbBr_3_ single‐crystalline film with similar results as the Bark group.^86^ This is also true for single‐crystalline MAPbI_3_ because they have similar electronic and carrier dynamic properties. However, to date, it is difficult to prepare thin single‐crystalline MAPbI_3_ films at the 5 µm scale.

To overcome the drawbacks of this high intrinsic resistance, the Huang group designed and prepared a lateral structured single‐crystalline perovskite solar cell (Figure 6e,f).^128^ Instead of collecting carriers in the vertical direction (carriers need to be transported through a thick absorber layer), the carrier was harvested in the lateral direction, during which the charge transport length is reduced, and the collection efficiency could be effectively enhanced, giving a PCE of 1.88%. However, Au electrodes were used for both the cathode and anode. The p–i–n regions were created by electric polling, which will introduce defects or crystal distortions, increasing the carrier recombination. It is reasonable to expect that the PCE of the lateral single‐crystalline perovskite solar cell could be further improved using more efficient hole and electron transport layers. Recently, the Sung group developed a geometrically confined lateral crystal growth method to prepare wafer‐scale single‐crystalline perovskite film. By using a more efficient electron collection electrode, PCBM/Ag and Au electrode, a lateral solar cell based on single‐crystalline perovskite achieved a PCE up to 4.83%.^111^ Since there are gaps between the single‐crystalline perovskite strips reported in this work, improving the coverage of single‐crystalline perovskite film is helpful for device performance.

#### 2.3.2 Photodetectors

Photodetectors have the potential to transform background electromagnetic waves into electronic signal and provide useful and important data for automatic control, information transportation, medical diagnosis, etc. Depending on how the generated carriers are utilized, current photodetectors can be classified as a photoconductive or photovoltaic. Next, the basic mechanism of single‐crystalline photodetector devices will be introduced.

**Figure 7:**
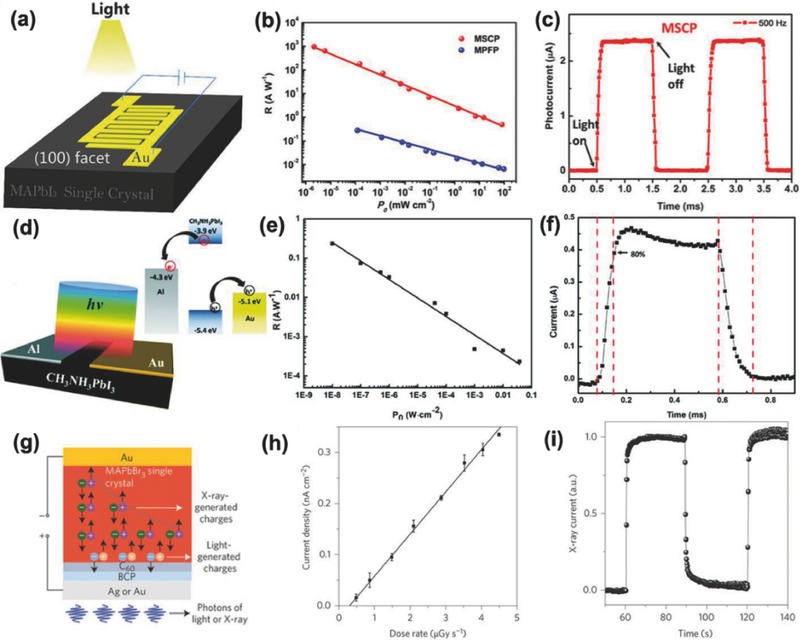
The single‐crystalline perovskite‐based photodetectors and their performances: a) a photoconductive photodetector with a single‐crystalline perovskite as the working media and b) its corresponding responsivity and c) response speed. Reproduced with permission.^81^ Copyright 2015, published under the terms of CC‐BY 4.0 license. d,g) a photovoltaic photodetector with an asymmetrical electrode to collect photogenerated holes and electrons and their corresponding device performance e, f, h, i). d–f) Reproduced with permission.^129^ Copyright 2016, RSC. g–i) Reproduced with permission.^106^ Copyright 2016, Nature Publishing Group.

*Photoconductive Detectors*: In a photoconductive photodetector, symmetrical electrodes (**Figure** **7**a), such as Au‐material‐Au, Ag‐material‐Ag, or ITO‐material‐ITO, are deposited onto the working materials.^81^, ^85^, ^108^, ^122^, ^123^ While working, the device current is monitored by applying a voltage bias on the symmetrical electrode, which is an important parameter to evaluate the device performance. According to Ohm's law (*I* = *V*/*R*), a variation of resistance will result in current changes. Turning the light on and off will make the carrier concentration of the device vary dramatically, which will result in a large difference in device resistance (*R*) and corresponding current (*I*) (Figure 7b). This process will generate a pulse‐like pattern in the *I*–*t* curve, as shown in Figure 7c. This will make the electromagnetic wave signal detection possible. A bias voltage is always needed for this type detector.

For single‐crystalline perovskites (Table 1), the intrinsic carrier concentration is very low (≈10^9^ cm^−3^). This is a major reason for the observed large resistivity (small conductivity approximately 1 × 10^−8^ Ω^−1^ cm^−1^). After being excited by photons, the carrier concentration will increase dramatically, which will alter the resistivity of the single‐crystalline perovskite. This results in a large difference in device current, which could allow single crystals to be used in photoconductive detectors. Equation (4) shows that the responsivity of a photodetector is closely related to the carrier concentration variations between the light and dark condition

(4) $$R\;\;=\;\;\frac{I_{\mathrm{ph}}\;\;-\;\;I_{\mathrm{dr}}}{P_{\mathrm{in}}\;\;\times \;\;s}\;\;\propto \;\;\frac{n_{\mathrm{ph}}\;\;-\;\;n_{\mathrm{dr}}}{P_{\mathrm{in}}\;\;\times \;\;s}$$

Here, *R* is the responsivity, *I*_ph_ and *I*_dr_ are the current of device under light illumination and dark, *P*_in_ is the power of the incident photons, *s* is the effective area of device, and *n*_ph_ and *n*_dr_ are the carrier concentration in light and dark, respectively. Under the same photocarrier generation rate with a lower intrinsic carrier concentration, larger responsivity values are seen for the photodetector. From this point of view, the single‐crystalline perovskites are great candidates for photodetector applications due to their extremely low intrinsic carrier concentration. Using the simple lateral electrode configuration described in Figure 7a, a MAPbI_3_‐based device achieved a responsivity as high as 953 A W^−1^ and an EQE as large as 2.22 × 10^5^% under a 1 V bias voltage and 2.12 nW cm^−2^ 532 nm light illumination.^81^ The single‐crystalline photodetector showed ≈100 times higher responsivity and EQE than its polycrystalline counterparts under 1 mW cm^−2^ light illumination. In addition to high responsivity and EQE, the simple‐structured photodetector also shows a very fast response time, rise time, and decay time, with values as small as 74 and 58 µs, respectively; other groups have reported similar results (**Table** **2**).

**Table 2:** Summary of photodetection properties of single‐crystalline perovskite‐based detectors

Materials	Device structure	Responsivity	Speed	EQE
(100) facet of MAPbI_3_ single crystal^81^	Au interdigital electrodes	953 A W^−1^	Rise: 74 µs Fall: 58 µs	2.22 × 10^5^ 1 V bias, with 532 nm light illumination 2.12 nW cm^−2^
MAPbI_3_ 129	Au–Al asymmetric electrodes	0.24 A W^−1^ 365–808 nm	Rise: 71 µs Fall: 112 µs	
MAPbI_3_ 130	Au interdigital electrodes	7.92 A W^−1^ 196 V (mW cm^−2^)^−1^	0.2 s	White light illumination
MAPbI_3_ (Cl, Br and Br, I mixture)^45^	Au–Ga asymmetrical electrodes	Narrow band selectivity		
MAPb (Br*_x_*I_1−_ *_x_*)_3_ 123	Au interdigital electrodes	2.4 A W^−1^ (460 nm, 2 V bias, 0.055 mW cm^−2^)	Rise: 3.4 ms Fall: 3.6 ms	639 (460 nm, 2 V bias, 0.055 mW cm^−2^)
MAPbBr_3_ 85	ITO electrodes		25 µs	
MAPbBr_3_ 131	Pt/crystal/Au asymmetrical electrodes		Rise: 70 µs Fall: 150 µs	
MAPbCl_3_ 68	Pt/crystal/Ti/Au asymmetrical electrodes	46.9 A W^−1^	Rise: 24 ms Fall: 62 ms	

*Photovoltaic Detectors*: Photoconductive detectors can work without a bias voltage. A photovoltaic detector is simply a solar cell device, in which the asymmetrical electrodes causing a built‐in electric field assist the collection of a photon‐generated carrier. The asymmetrical electrode (Figure 7d,g) could form p–i–n type, p–n type, or Schottky‐type junctions to assist with carrier collection. Theoretically, no bias voltage is needed for this type of device. The Yan group built a planar Schottky‐junction type detector by depositing asymmetrical Au and Al electrodes, which can facilitate hole and electron collection at each electrode.^129^ A PCE of 0.79% was obtained via this simple device. This device can achieve a responsivity of 0.24 A^−1^ under 0 V bias and 1 × 10^−8^ W cm^−1^ 808 nm light illumination. The single rise and decay times were 71 and 112 µs, respectively (Figure 7f).

The Huang group first developed Au/mix‐halide perovskites/Ga structured p–i–n type detectors,^42^ which show a narrow bound response due to a high surface recombination rate. They further optimized single crystallinity, which resulted in a hole and electron mobility as high as ≈217 and 206 cm^2^ V^−1^ s^−1^, as well as a high µτ product of 1.4 × 10^−2^ cm^2^ V^−1^ and low surface recombination rate of 64 cm s^−1^. They developed a p–i–n type X‐ray detector with a configuration of Au/MAPbBr_3_/C_60_/BCP/Ag or Au‐similar to traditional solar cell structures (Figure 7g,h).^106^ As a photodetector, this device shows a very small noise‐equivalent power of ≈10 pW cm^−2^, indicating that this device can be used to detect a very weak light signal. The authors further utilized this device as an X‐ray detector because of its high µτ product and lead element, which is believed to be an index for good X‐ray detectors. The current generated from the X‐ray increased linearly with the X‐ray dose rate. A sensitivity of 80 µC Gy^−1^_air_ cm^−2^ was derived, which is more than 10 times higher than that of a Cd (Zn) Te single‐crystal X‐ray detector and 4 times higher than that of the currently used α‐Se X‐ray detector. The lowest detectable dose was as low as 0.5 µC Gy_air_ s^−1^, which is lower than required by regular medical diagnostics. This new X‐ray detector could reduce the radiation dose during medical and security checks, with a very fast response speed of approximately 730 µs.^106^

#### 2.3.3 Lasers

Hybrid perovskites are high‐gain materials for lasing because of their high absorption coefficient, high photoluminescence quantum yield, slow Auger recombination rate, long carrier diffusion length, low defect density, etc. Sandwiched between spiro‐OMeTAD and TiO_2_, a polycrystalline MAPbI_3_ film still shows a strong amplified spontaneous emission, demonstrating its high optical gain.^134^ In addition to the high‐gain material, a cavity is also needed to achieve population inversion. Using a gold film and a dielectric film stack, the Snaith group produced a polycrystalline perovskite film laser with a threshold of 200 nJ cm^−2^.^134^ While this result is promising, the full width at half maximum (FWHM) of the laser emission is small and produced a larger *Q* factor. This may be a result of the trap state generated by the grain boundaries, which will broaden the photoluminescence.

**Figure 8:**
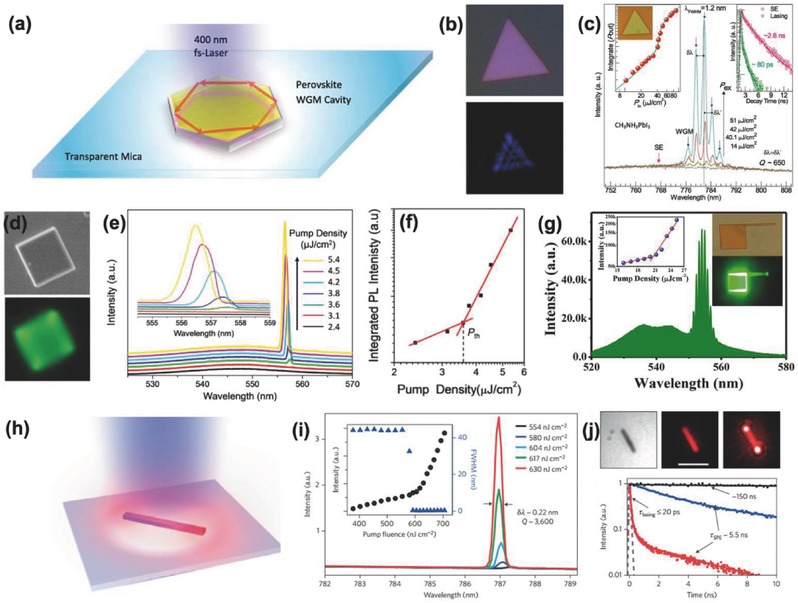
Laser emission from perovskites with special shapes: hexagonal plate, microdisk, and nanowire; a) the working mechanism of a WGM type cavity produced by perovskite crystals and its laser emission properties (b, c). Reproduced with permission.^132^ Copyright 2014, ACS. d) Microdisk‐based WGM cavity achieving laser emission and its threshold properties (e, f); g) nanowire‐coupled microdisk producing directional laser emission. Reproduced with permission.^83^ h) Schematic illustration of laser emission from a perovskite nanowire, which naturally forms a FP cavity to support laser emission and laser emission properties (i, j). Reproduced with permission.^133^ Copyright 2015, Nature Publishing Group.

To overcome the drawbacks of grain boundaries, high‐quality perovskite nanoplates have been prepared using a two‐step CVD method and used as a gain material.^132^ In contrast to Snaith's report, in which an Au film and dielectric film stack served as a cavity, and the boundary of the nanoplate forms a whispering‐gallery‐mode (WGM) cavity that can utilize successive total internal reflection and provide a high *Q* factor. Using a MAPbI_3_ nanoplate, they can achieve 780 nm laser emission with a threshold of 37 µJ cm^−2^ and *Q* factor of ≈650 (**Figure** **8**a–c). With the same strategy, Liao et al. built a single‐crystalline microdisk MAPbBr_3_ laser with an emission of 557.5 nm, threshold of 3.6 µJ cm^−2^, and a *Q* factor of 430 (Figure 8d–g).^83^ However, the laser direction could be controlled in this system. In addition, the Song group developed a way to prepare a single‐crystalline MAPbBr_3_ microdisk with a wire ‘antenna' that can direct the laser emission.^135^ This is good for micro‐optoelectronic devices. Except for the WGM cavity that intrinsically possesses the disk/plate shape device, the two surfaces of a nanowire could serve as Fabry–Pérot (FP) cavity for laser emission. The Zhu and Song group have developed MAPbI_3_, MAPbBr_3_, and MAPb(I_1−_ *_x_*Br*_x_*)_3_ nanowire lasers with an even smaller threshold of ≈1 µJ cm^2^ and a *Q* factor exceeding 2000 (Figure 8h–j).^133^

## 3 Summary

Perovskite solar cells have recently achieved exponentially increasing PCEs. In addition to optimizing the electron transporting layer and hole transport layer (HTL), the successive improvements of perovskite film quality, such as film coverage, grain size, and surface passivation, offer more incremental PCE improvements. The basic properties, including the charger carrier lifetime, light absorption region, and carrier diffusion length, single‐crystalline perovskites have shown great advantages in laser and photodetector applications and have the potential to be better candidates for solar cell applications. However, there are few reports on highly efficient single‐crystalline perovskite solar cells. To further obtain high efficiency single‐crystalline solar cells, three problems should be considered: the large light absorption coefficient, the intrinsic low conductivity, and the device structure. The large light absorption coefficient determines that the position of the photon‐generated carriers is close to the top surface of the light incident side. To successfully extracted the carriers, (1) a thin single‐crystalline perovskite film (10 µm scale) should be prepared; (2) thick perovskite films should be doped to make the carrier concentration high enough, which can reduce the ohmic loss during the carrier collection in a thick single‐crystalline perovskite film; (3) the interface should be manipulated to achieve increased electronic contact, as well as less defects; and (4) in addition to material optimization, new device structures, such as lateral design, should be applied for special morphologies and electrical properties. These great optoelectronic properties also endow perovskite materials with potential application in high‐speed photon detection because of their high carrier mobility and long carrier lifetime, highly sensitive X‐ray or γ‐ray detection because of Pb, as well as large mobility and lifetime product, efficient lasers, etc. Further developments in these applications should be focused on: (1) device integration to achieve a highly efficient X‐ray detection system to alleviate bio‐damage during safety and medical examination and (2) integrating perovskite crystals into a laser cavity to achieve a low threshold for an energy efficient laser device with whole spectrum coverage.

## Conflict of Interest

The authors declare no conflict of interest.
